# Metformin can block precancerous progression to invasive tumors of bladder through inhibiting STAT3-mediated signaling pathways

**DOI:** 10.1186/s13046-015-0183-0

**Published:** 2015-08-07

**Authors:** Qi Pan, Guo-Liang Yang, Jiang-Hua Yang, Shi-Long Lin, Ning Liu, Shan-Shan Liu, Meng-Yao Liu, Lian-Hua Zhang, Yi-Ran Huang, Ru-long Shen, Qiang Liu, Jian-Xin Gao, Juan-Jie Bo

**Affiliations:** Department of Urology, Renji Hospital, School of Medicine, Shanghai Jiao Tong University, Shanghai, China; State Key Laboratory of Oncogene and Related Genes, Shanghai, China; Laboratory of Tumorigenesis and Immunity, Clinical Stem Cell Research Center, Renji Hospital, Shanghai Jiao Tong University School of Medicine, Shanghai, China; Department of Pathology, Renji Hospital, School of Medicine, Shanghai Jiao Tong University, Shanghai, China; Department of Pathology, Ohio State University School of Medicine, Columbus, OH USA

**Keywords:** Metformin, Preneoplasm, Bladder cancer, Stat3, Rat, Cell cycle, Apoptosis, Migration, Invasion

## Abstract

**Background:**

Metformin is the first line of oral antidiabetic drug in the biguanide class for treatment of type 2 diabetes. Increasing evidence has suggested that it is a potential anti-tumor drug. However, the mechanisms underlying inhibiting tumor development remain elusive, especially in bladder tumors.

**Methods:**

T24 and J82 cell lines were used as an in vitro model, and 24 female SD rats were used to build an N-methyl-N-nitrosourea (MNU)-induced orthotopic rat bladder cancer model. Transfection of lentivirus-based shRNA was used to construct the STAT3-KNOCKDOWN T24 cell line. After metformin treatment, the viability of bladde cancer cells was determined by CCK8. Cell cycle distribution and apoptosis were assessed by flow cytometry. The migration and invasion abilities of cells were evaluated by wound healing and transwell asssays. The inactivation of stat3 pahtway was examined by qRTPCR, western blot and Immunofluorescence.

**Results:**

Metformin can effectively inhibit precancerous progression to invasive cancer in an MNU-induced rat orthotopic bladder tumor model, although it could not completely suppress normal cells transforming into tumor cells. While the MNU could induce 50 % rats (4/8) to develop invasive bladder cancers, the rats co-administrated with metformin failed to develop invasive tumors but retained at precancerous or non-invasive stages, exhibiting as dysplasia, papillary tumor and/or carcinoma in situ (CIS). Accordingly, phosphorylation of signal transducer and activator of transcription 3 (STAT3), which is a well known oncogene, was significantly inhibited in the tumors of rats treated with metformin. *In vitro* experiments revealed that the metformin could efficiently inhibit STAT3 activation, which was associated with the cell cycle arrest, reduction of cell proliferation, migration and invasiveness, and increase in apoptotic cell death of bladder cancer cell lines.

**Conclusions:**

These findings provide for the first time the evidence that metformin can block precancerous lesions progressing to invasive tumors through inhibiting the activation of STAT3 pathway, and may be used for treatment of the non-invasive bladder cancers to prevent them from progression to invasive tumors.

**Electronic supplementary material:**

The online version of this article (doi:10.1186/s13046-015-0183-0) contains supplementary material, which is available to authorized users.

## Introduction

Bladder cancer is the most common malignancy of the urinary tract, ranking the 7th most common cancer in men and the 17th in women [1]. At initial diagnosis, nearly 75–80 % of cases are Non-Muscle-Invasive Bladder Cancer (NMIBC, pTa-pT1), with the remaining Muscle-Invasive Bladder Cancer (MIBC) [2]. The standard therapy for NMIBC is Transurethral Resection of Bladder Tumor (TURBT) combined with subsequent intravesical therapy, with a high 5-year survival rates [2]. However, the five-year recurrence and progression rates can add up to 31–78 % and 1–45 % respectively after the first treatment [3, 4]. Therefore, finding an effective way of preventing NMIBC progression and recurrence is required urgently.

Metformin, 1, 1-Dimethylbiguanide hydrochloride, is one of the most popular drugs which are used for type 2 diabetes therapy. It works by targeting the enzyme AMP activated protein kinase (AMPK), which is regulated by a protein kinase known as Liver Kinase B1 (LKB1), and inducing muscles to take up glucose from the blood [5]. The LKB1 is a well recognized tumor suppressor and can be activated by exercise, suggesting that metformin might participate in suppressing tumorigenesis [6]. A growing body of epidemiological investigation and preclinical studies has shown that metformin treatment is associated with reduced risk and better prognosis of various types of cancers [6–8]. Interestingly, some recent retrospective studies have demonstrated that metformin could exert protective effects on recurrence in NMIBC patients and cancer-specific mortality in MIBC patients treated with radical cystectomy [9, 10]. However, the effects of metformin on bladder cancer have not been adequately investigated and, in particular, the mechanisms underlying the metformin inhibiting bladder cancer remain to be elucidated.

Signal transducer and activator of transcription 3 (STAT3) is a cytoplasmic transcription factor that can be activated by tyrosine phosphorylation at position 705, causing its dimerization, nuclear translocation, DNA binding, and activation of gene transcription [11]. It is known that STAT3 is abnormally activated in many cancers and plays an important role in tumor proliferation and metastasis. It regulates cellular proliferation, invasion, migration, and angiogenesis [11, 12]. Moreover, it has been reported that activation of STAT3 is crucial for bladder cancer carcinogenesis, growth, survival and progression *in vitro* and *in vivo* [13, 14]. STAT3 has been considered as a promising molecular target for cancer therapy.

The purpose of this study is to evaluate the effects of metformin on bladder cancer using an *in vitro* model of human urinary bladder-cancer and an *in vivo* model of rat orthotopic bladder cancer and explore the role of metformin in regulating STAT3 pathway.

## Materials and methods

### Cell lines, medium and cell culture

Human bladder cancer cell lines T24 and J82 were purchased from the American Type Culture Collection (ATCC, Rockville, MD, USA) and were cultured in 10 % fetal bovine serum (Invitrogen) Dulbecco’s Modified Eagle’s Medium (DMEM) (Invitrogen, Carlsbad, CA, USA)) supplemented with penicillin (100 units/ml) and streptomycin (100 μg/ml). Cells were incubated at 37 °C with 5 % CO_2_.

### Construction of STAT3-KD Cell Line

To construct a stable STAT3-KNOCKDOWN cell line, we transfected T24 cells with lentivirus-based shRNA vector (purchased from GenePharma, Shanghai, China). The shRNA oligonucleotides sequences targeting STAT3 and acting as normal control are as follows: GCGTCCAGTTCACTACTAAAG; TTCTCCGAACGTGTCACGT. Transfections were performed with polybrene (GenePharma) according to manufacturer’s instruction. Stable clones were selected in 1000 μg/ml neomycin (Invitrogen) for 2 months.

### Cell viability assay

Cell viability assays were performed with a Cell Counting Kit-8 (Dojindo, Kumamoto, Japan). Cells were seeded in 96-well plates in triplicate (5 × 10^3^ per well) for 24 h. Then the medium was removed and replaced by fresh culture medium containing metformin (Sigma-Aldrich, St. Louis, MO, USA) in various concentrations (0, 10, 20, 40 or 60 mM) for 24 or 48 h. The number of viable cells per well was measured by the absorbance (450 nm) of reduced 2-(2-methoxy-4-nitrophenyl)-3-(4-nitrophenyl)-5-(2, 4-isulfophenyl)-2H-tetrazolium (monosodium salt) using the Microplate Autoreader (Bio-Tek Instruments Inc., Winooski, VT, USA). Independent experiments were repeated for three times.

### Analysis of cell cycle and apoptosis

Cell apoptosis detection kit (propidium iodide (PI), RNase staining buffer and FITC-labeled Annexin V) were purchased from BD Pharmingen (San Diego, CA, USA). Cells were seeded 2.5 × 10^5^ per well in 6-well plates for 24 h. Then the medium was replaced by culture medium containing metformin 0, 20 or 40 mM for 24 or 48 h. The cells were harvested for analysis of cell cycle and apoptosis, respectively. The cell cycle was analyzed using PI staining, according to the manufacturer’s instructions. Briefly the cells were fixed in 70 % ethanol, stained with PI, and the amount of PI-labeled DNA in a cell was measured by a flow cytometer (Accuri C6, Becton Dickinson, San Jose, CA, USA). The acquired data were analyzed by FlowJo software (Ashland, OR, USA). To determine the apoptotic cells, the cells were stained with Annexin V-FITC and PI immediately after harvesting, and analyzed by flow cytometry, as described by the manufacturer’s instructions.

### Wound healing assay

T24 cells were seeded 5 × 10^5^ per well in 6-well plates and cultured until they reached complete confluence. Cells were scratched with a pipette tip and washed with PBS buffer. Then cells were cultured in 1 % FBS DMEM containing metformin (0, 10 or 20 mM). Photographs were taken in pre-marked spots at 0, 12 and 24 h of culture for comparison. The number of cells migrated into the wound areas was counted.

### Transwell assay

T24 cells were treated with metformin (0, 10 or20 mM) for 24 h. Then cells were seeded 3 × 10^4^ cells per well in 150 μl 1 % FBS DMEM supplemented with 0, 10 or 20 mM metformin into the upper chamber of the transwell in 24-well plates (growth surface area of insert: 0.33 cm^2^; membrane pore size, 8 μm; Corning Incorporated; Corning, NY, USA) with or without Matrigel (BD Pharmingen), and 500 μl 10 % FBS DMEM was added into the lower chamber. After 24 h, the bottom of the inserts were fixed in methanol for 10 min and stained with 0.1 % crystal violet staining solution. The cells migrating or invading into the bottom-lower surfaces of inserts were measured by using an inverted phase contrast microscope.

### Western blot analysis

The cells and tissues were collected and lysed in Radio-Immunoprecipitation Assay (RIPA) buffer (Thermo Fisher, Rockford, IL, USA) containing 1 % phenylmethylsulfonyl fluoride (PMSF). Protein concentration was quantified with the BCA Protein Kit (Beyotime, China). Lysates (30 μg protein) were separated by 10 % SDS-PAGE gels electrophoresis and transferred to a PVDF membrane (Bio-Rad, Hercules, CA, USA). The membranes were blocked in TBS/Tween20 (TBST) buffer containing 5 % non-fat milk powder for 2 h at room temperature, and then probed with primary antibodies overnight at 4 °C. Rat monoclonal antibodies (mAbs) to pstat3 (Y705), stat3, cyclin D1, Bcl-XL, Bcl2 and β-actin were purchased from Cell Signaling (Beverly, MA, USA), horseradish peroxidase (HRP)-conjugated anti-rabbit IgG antibody was purchased from Santa Cruz Biotechnology (Santa Cruz, CA, USA). After incubated with secondary antibodies for 1 h at room temperature, protein signal was detected with the ECL chemiluminescent detection system (Bio-Rad), and protein levels were normalized to β-actin.

### Immunofluorescent microscopic analysis

For cytochemical analysis of phosphorylated STAT3 (pstat3), cells cultured in 96-well plates were fixed and permeablized in methanol for 30 min followed by blockage in 5 % bovine serum albumin (BSA) for a another 30 min and then incubated with primary antibody overnight at 4 °C. The cells were washed 3 times with Tris-Buffer Solution Tween (TBST) and then incubated for 1 h with FITC-conjugated anti-rabbit IgG secondary antibody (BD Pharmingen) at room temperature. Nuclei were stained with Hoechst 33342 (10 μg/ml; Sigma-Aldrich) for 3 min. Samples were examined and microphotographed under a fluorescent microscope (Nikon Instruments Inc. Melville, USA).

### Real-time PCR analysis

Total RNA was extracted from human bladder cancer cells using Trizol (Invitrogen). The cDNA was synthesized from 0.5 μg of RNA with a Prime Script Kit (TAKARA, Toyobo, Osaka, Japan). Gene transcription levels were quantified by real-time quantitative PCR with SYBR Green PCR real-time PCR Master Mix (TAKARA). β-actin was used as an endogenous control. All the samples were detected in triplicate for each experiment. All results shown were representatives of three independent experiments, and mRNA levels were expressed as 2^-ΔΔCT^. The PCR were performed with a two-step qRT-PCR at 95 °C for 10 min, then 40 cycles of 95 °C for 15 s and 60 °C for 1 min, followed by 95 °C for 15 s. The specific primers used were listed as follows:

- β-actin forward: CATGTACGTTGCTATCCAGGC, reverse: CTCCTTAATGTCACGCACGAT,
- Bcl2 forward: GGTGGGGTCATGTGTGTGG, reverse: CGGTTCAGGTACTCAGTCATCC.
- Bcl-XL forward: GAGCTGGTGGTTGACTTTCTC, reverse: TCCATCTCCGATTCAGTCCCT.
- cyclin D1 forward: GCTGCGAAGTGGAAACCATC, reverse: CCTCCTTCTGCACACATTTGAA.

### Establishment of orthotopic rat bladder cancer model

Twenty-four female SD rats weighting around 280 g were obtained from the Experimental Animal Center of Renji Hospital, School of Medicine, Shanghai Jiaotong University (Shanghai, China) and housed in the center. Animal protocols in the study were approved by the Institutional Animal Care and Use Committee of Renji Hospital, School of Medicine, Shanghai Jiaotong University. They were maintained in an air-conditioned room lighted 12 h every day , given standard laboratory rat chow and could freely access to tap water. All rats were acclimatized for 7 days before the experiments started. The rats were divided into three groups (Additional file 1: Figure S1): a control group (*n* = 8); an N-methyl-N-nitrosourea (MNU)-treated group (MNU group) (*n* = 8); and a group of rats cotreated with MNU and metformin (Met group) (*n* = 8). Briefly, the rats were anesthetized i.p. with Nembutal (50 mg/kg). Two mg of MNU (Sigma-Aldrich) that was dissolved in sodium citrate buffer (10 mg/ml) was administered to the rats of the MNU group and the Met group via rat epidural catheter intravesically within 30 min of preparation of MNU solution every other week (weeks 0, 2, 4 and 6) for a total 4 doses. The rats remained anesthetized for approximately 2 h after catheterization. Rats of the Met group were administered with metformin (2 g/L) in the drinking water. All the rats were monitored for 14-weeks. Body weight (BW) was measured every two weeks and drinking water was replaced twice a week during the experimental period.

**Fig. 1:**
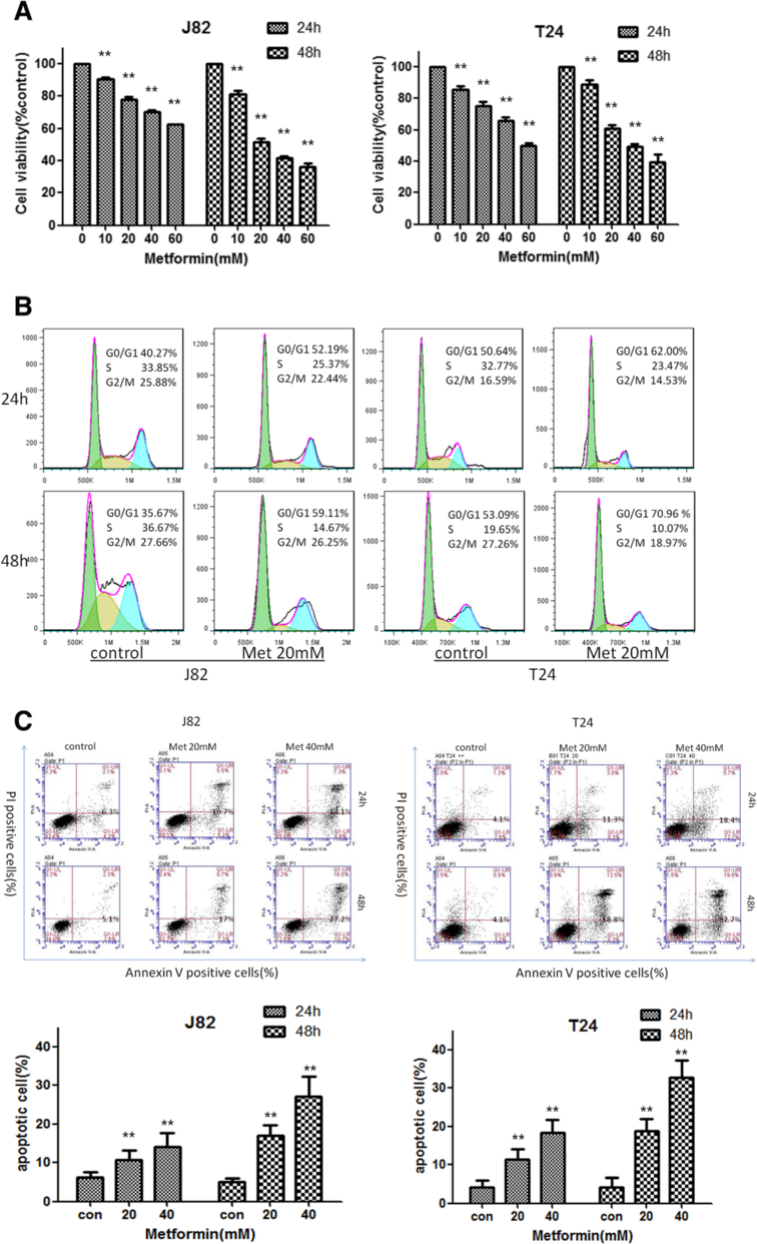
Metformin inhibited the proliferation, cell cycling and viability of bladder cancer cells. **a** *Inhibition of cell proliferation:* J82 and T24 cells were seeded 5 × 10^3^ per well in 96-well plates for 24 h. Then cells were treated with metformin (0, 10, 20, 40, or 60 mM) for 24 or 48 h. Cell numbers and viability was evaluated by CCK8. **b** *Cell cycle arrested at G*_*0*_ */G*_*1*_ *phases:* J82 and T24 cells were treated with 20 mM metformin for 24 or 48 h and cell cycle were analyzed by flow cytometry. **c** *Promotion of apoptotic cell death:* J82 and T24 cells were treated with 20 and 40 mM metformin for 24 or 48 h, stained with PI and FITC-labelled Annexin V and determined by flow cytometry for the frequency of apoptotic cells. Top panel: representative histograms of cell cycling; bottom panel: summary of apoptotic cells (%) from three reproducible experiments. **, *P* < 0.01 when compared to control group (0 mM) in all the experiments

At the end of the experiment, all the rats were sacrificed by intraperitoneal administration of an overdose of Nembutal. The lungs, stomach, liver, kidneys, uteruses and intestines were harvested and evaluated for metastatic lesions. Before removal, bladders were fixed by intravesically injecting 1 ml buffered formaldehyde solution. After overnight pre-fixation, each bladder was cut open, and its mucosal surface was macroscopically evaluated for urothelium lesions. Then the bladder tissues were processed for paraffin-embedding and sectioning. The paraffin-embedded tissues were sectioned in a 2 μm thickness, mounted in slides and stained with haematoxylin & eosin (H&E). The slides were randomized and carefully examined histologically by two pathologists indepently. Histologic lesions were classified and staged according to the World Health Organization/International Society of Urological Pathology Consensus Classification of Urothelial (Transitional Cell) Neoplasms of the Urinary Bladder.

### Statistical analysis

All data were presented as means ± S.D. All statistical analysis was performed with GraphPad Prism version 5.00 software from GraphPad Software (San Diego, CA, USA). For *in vitro* studies, comparison between control group and metformin-treated group was performed by using Student’s t-test. For *in vivo* studies, difference between tumor areas was assessed by Student’s t-test, and histopathological examination was evaluated by Chi-square test. The P-value of < 0.05 was considered statistically significant, *p* < 0.01 was considered statistically highly significant.

## Result

### Metformin inhibits the proliferation and expansion of bladder cancer cells by inducing cell cycle arrest and apoptosis, respectively

To evaluate the effects of metformin on human bladder cancer cell lines, we treated two bladder cancer cell lines, J82 and T24, with metformin. Both cell lines were seeded in 96-well plates and cultured in the presence of metformin at variable concentrations (0, 10, 20, 40 or 60 mM) for 24 and 48 h. Then cell viability was determined by CCK8 assay. Metformin is capable of inhibiting the growth of both J82 and T24 cell lines in a dose—and time-dependent manner, as shown in Fig. 1a. To determine the underlying mechanism, we further investigated the effect of metformin on the cell cycling of the bladder cancer cells by flow cytometry. Treatment of J82 and T24 cells with 20 mM metformin for either 12 or 24 h arrested cell cycle at the G_0_/G_1_ phase. As shown in Fig. 1b, a significantly increased proportion of cells was observed arrested at the G_0_/G_1_ phase, while the percentage of cells in the S phase sharply decreased accompanied by a drop in the percentage of cells at the G_2_/M phase. Whether metformin can induce apoptosis in bladder cancer cells was also determined by flow cytometry. We treated both cell lines (J82 and T24) with 0, 20 or 40 mM metformin for 24 and 48 h and then analyzed their apoptosis by flow cytometry. The result showed that metformin induced an increase in the percentage of apoptotic cells in both cell lines in a dose—and time-dependent manner (Fig. 1c). These results demonstrate that metformin inhibits the proliferation of bladder cancer cell lines by blocking cell cycle progression at the G_0_/G_1_ phase and cell expansion by inducing apoptotic cell death.

### Metformin suppresses bladder cancer cell migration and invasion *in vitro*

**Fig. 2:**
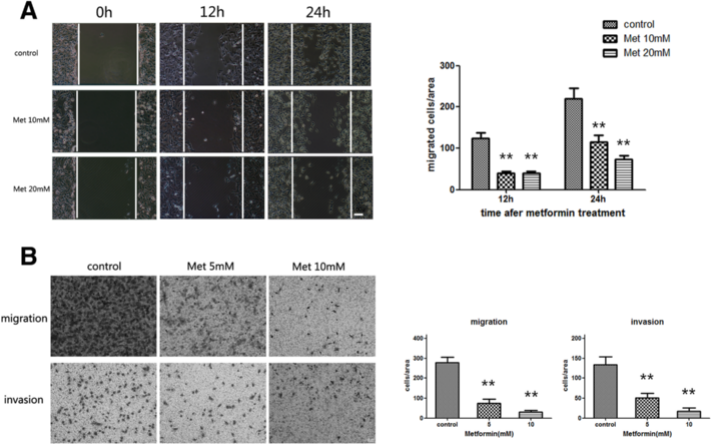
Metformin inhibited migration and invasion of bladder cancer cells. T24 cells were treated with metformin for 24 h, and the capacity of migration and invasion were assessed by wound healing assay (**a**) and transwell migration assays (**b**). Representative pictures of migration and invasion assays are shown in left panel and quantitative results are shown in right panels. The data presented are expressed as mean ± SD from 3 independent experiments. **, *P* < 0.01 when compared to control groups. Scale bars in the micrographs represent 100 μm

T24 cell line was derived from an invasive high-grade urothelial carcinoma [15], and so we selected T24 to evaluate the inhibitory effect of metformin on cell migration and invasion of bladder cancer with the wound healing assay as well as transwell assay. We treated T24 cells with 10 or 20 mM metformin, and compared the number of migrated or invaded cells with control groups. As shown in Fig. 2a, metformin significantly suppressed T24 cell migration to the wound area and the highest inhibitory effect was observed at 24 h in the wound healing assay. Consistently, the migration and invasion of T24 cells were also dramatically inhibited by metformin at 24 h of Transwell assay (Fig. 2b). Taken together, these results suggest that metformin can suppress the capability of bladder cancer cells to migrate and invade *in vitro*.

### Metformin can inhibit precancerous progression of rat bladder tumor

**Fig. 3:**
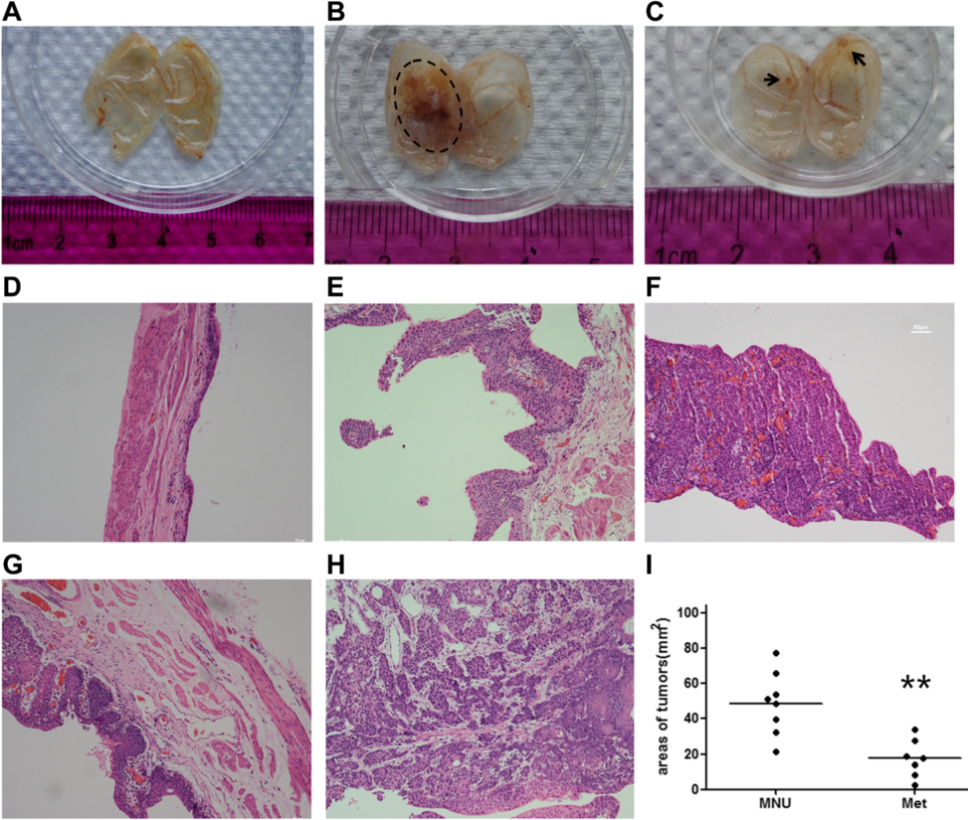
Macroscopic and microscopic examinations of tumors in MNU-induced and metformin-treated rat bladders. *Macroscopic graphs (A-C):* **a** Bladders from the control group showed a normal appearance; **b** Bladders from the MNU-induced group had thicker walls and a wider range of tumor lesions (outlined by the dotted line); **c** Bladders from the metformin-treated group showed some tiny and isolated lesions (indicated by the arrow). *Histopathological examinations (D-H):* tissue or tumor sections were stained with H & E and analyzed by pathologists double blindly. **d** Normal uroepithelial cells were observed in the control group; **e** Low grade and **f** high grade papillary tumor were found mainly in the metformin-treated group, and (**g**) CIS and (**h**) Infiltrative (invasive) tumors were found mainly in the MNU group. No invasive tumors were observed in Met group. **i** Summary of tumor sizes in the MNU group and Met group. **, *P* < 0.01 when compared to MNU group. Magnificaiton of micrographs: x100

Because metformin was observed to inhibit proliferation, viability, migration and invasion of bladder cancer cells *in vitro*, we next explored whether it had inhibitory effects on bladder carcinogenesis, using the MNU-induced orthotopic rat bladder cancer model. All rats were randomly divided into three groups (Additional file 1: Figure S1), including a control group, a MNU-induced group (MNU group) and a group of rats co-treated with MNU and metformin (Met group). All rats were survived at the end of the experiment and no significant difference in the body weight and diet consumption was observed among the three groups (data not shown). Macroscopical examination showed no neoplasmic lesions in the urothelial texture of bladders from the control group (0 %), but large neoplasmic lesions in the bladders from MNU-induced group (100 %; 8/8) and, interestingly, reduced neoplasmic lesions in frequency and size in the Met group (87.5 %; 7/8). The mean tumor surface areas per rat in the MNU group (48.84 ± 17.82; mm^2^; *n* = 8) was two times more (*P* < 0.05) than in the Met group (17.5 ± 10.88 mm^2^; *n* = 7), indicating a significant reduction of tumor growth in Met group (*P* < 0.05) (Fig. 3a-c & Table 1).

**Table 1:** The effects of metformin on the development of MNU-induced rat orthotopic bladder tumors

Macroscopy & microscopy	Control (*n* = 8)	MNU (*n* = 8)	MNU + Met (*n* = 8)	*P value*
Frequency of tumoringenivity [%(*n*/n)]	0	100(8/8)	87.5(7/8)	0.5
Areas of tumors [mm^2^(*n*)]	0	48.84 ± 17.82(in 8)	17.5 ± 10.88(in 7)	0.0014
Preneoplasic lesions[%(*n*/n)]				
Hyperplasia	0	0	0	1
Dysplasia	0	0	12.5(1/8)	0.302
Neoplasic lesions[%*n*/n]				
Papillary tumor	0	37.5(3/8)	75(6/8)	0.046
Carcinoma in situ	0	75(6/8)	37.5(3/8)	0.046
Infiltrative tumor	0	50(4/8)	0	0.021
Accompanied other pathology [%(*n*/n)]				
Squamous differentiation	0	37.5(3/8)	12.5(1/8)	0.248
Sarcoma	0	12.5(1/8)	0	0.302

*P* values represent comparion between MNU group and Met group

Note: Multiple lesions at various stages were detected in the same bladders of MNU-induced rats, including papillary tumors, CIS, infiltrative tumors and sarcomas. Upon treatment with metformin, MNU-induced rats were only detected with dysplasia, papillary tumors and CIS. Tumor incidence and tumor size (areas) in MNU-induced rats were reduced upon treatment with metformin

Histological examination further revealed that while various types of neoplastic lesions including precancerous and invasive ones occurred in the MNU-induced group, no invasive lesions were observed in the Met group (Fig. 3d-h & Table 1). As shown in Table 1, Met group developed significantly less cases of carcinoma in situ (CIS) (37.5 % vs 75 %, *P* < 0.05) and infiltrative (invasive) tumors (0 % vs 50 %, *P* < 0.05) than MNU group. At the time of sacrifice, precancerous lesions such as dysplasia were only observed in Met group, which had developed into CIS or infiltrative tumors in MNU group. Interestingly, four rat bladders from the Met group developed alone with papillary tumors, which always coexisted with CIS in the MNU group. CIS in the bladder is considered as an early stage of cancer with invasive potential, whereas papillary tumor is non-invasive with limited invasive potentials. The results strongly suggested that metformin was an efficient agent preventing progression of precancerous lesions to invasive tumors in the MNU-induced rat bladder cancers.

### Metformin blocks precancerous progression through inhibiting STAT3-mediated signaling pathways

**Fig. 4:**
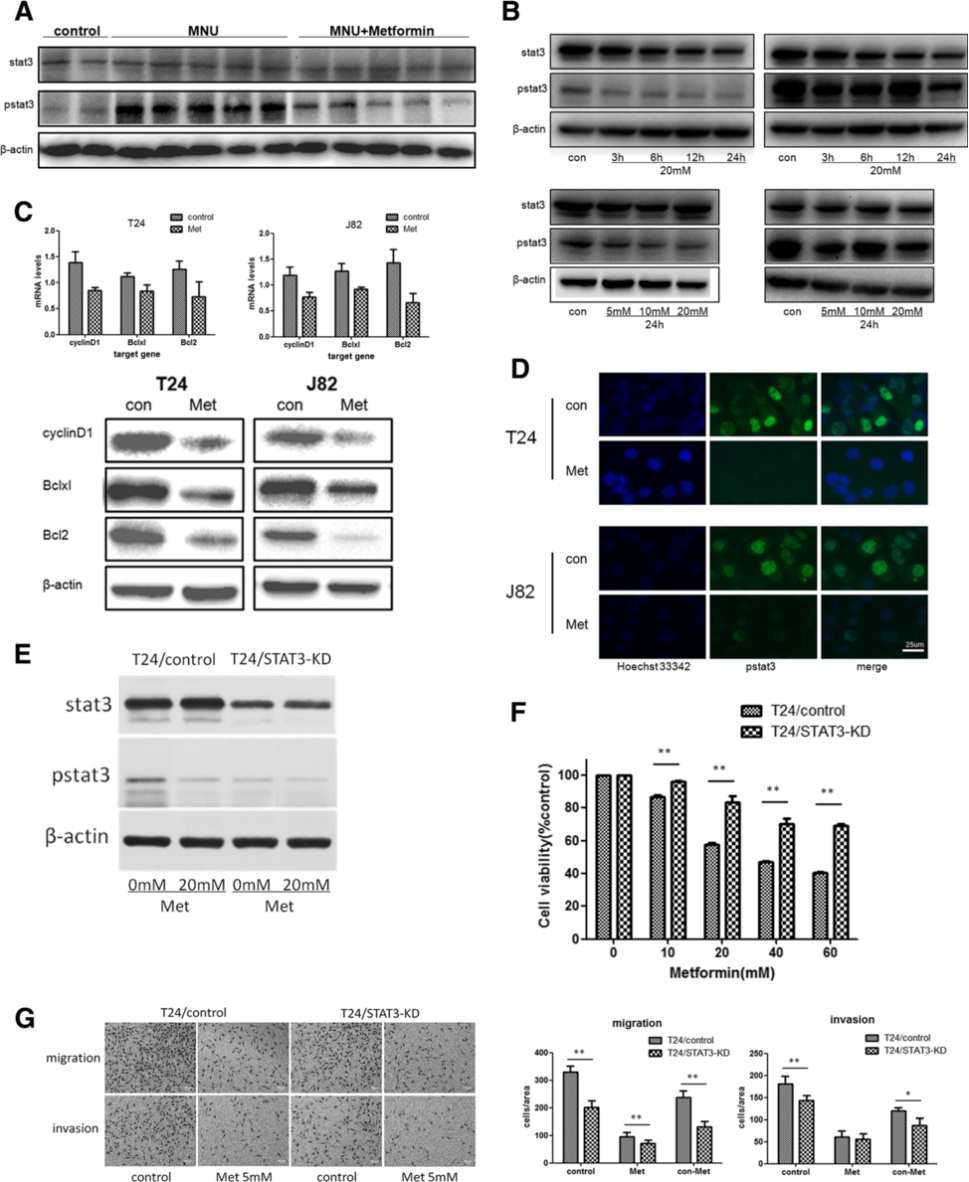
Metformin inactivated stat3-mediated signaling pathways in bladder cancer cells *in vivo* and *in vitro*. **a**-**b** The expression of STAT3 and pstat3 *in vivo* and *in vitro* with or without treatment with metformin were examined by western blot. **c** The expression of transcripts (top panel) and proteins (bottom panel) of STAT3 targeting genes in bladder cancer cell lines treated with or without metformin, including cyclinD1, Bcl-XL and Bcl2. Top panel: the transcripts assessed by qRT-PCR, bottom panel: the proteins detected by Western blot. **d** The expression and location of pstat3 in bladder cancer cell lines treated with or without metformin were evaluated by immunofluorescent microscopy. Scale bar represents 25 μm. **e** Construction of the T24/STAT3-KD bladder cancer cell line with the relative low level of phospholyrated STAT3 compared with normal control cell line T24/control. **f** T24/STAT3-KD and T24/control cells were treated with metformin (0, 10, 20, 40, or 60 mM) for 48 h. Cell viability was evaluated by CCK8. **g** T24/STAT3-KD and T24/control cells were treated with metformin for 24 h, and the abilities of migration and invasion were assessed by Transwell. Representative pictures of migration and invasion assays are shown in left panel and quantitative results are shown in right panels. Scale bars in the micrographs represent 100 μm. All data presented are expressed as mean ± SD from 3 independent experiments. **, *P* < 0.01; *, *P* < 0.05

It has been reported that STAT3 activation in urothelial basal cells results in progression of CIS to invasive cancer in an orthotopic mouse bladder cancer model [13]. Therefore, we investigated whether metformin suppressing progression of precancerous lesions to invasive cancers in the MUN-induced rat bladder cancer model was associated with inhibition of STAT3 activation. We harvested “normal” urothelial cells from control group and tumor tissues from the MUN and Met groups for Western blot analysis. The results showed that STAT3 phosphorylation was significantly activated in tumor tissues compared with normal urothelial cells. However, the activation was remarkably weakened in Met group, compared to MNU group (Fig. 4a), suggesting that metformin blocked progression of MNU-induced precancerous lesions to invasive ones through inhibiting STAT3 phosphorylation.

To confirm the finding, we next treated T24 and J82 cell lines with metformin and evaluated its effect on activation of STAT3 signaling pathways by western blotting and immunofluorescent microscopy. As shown in Fig. 4b and d, phosphorylated STAT3 was constitutively detected in the nucleus of the bladder cancer cell lines T24 and J82 without treatment with metformin. The level of phosphorylated STAT3 was remarkably decreased after treatment with metformin in dose—and time-dependent manners. Because STAT3 is a classical cancer-related transcription factor and regulates a range of downstream target genes such as cyclin D1, Bcl-XL and Bcl2 that play important roles in cell cycle and cell survival [11], we analyzed the expression of these genes at the levels of mRNA and protein in the cells treated with or without metformin. Upon the treatment with 20 mM metformin for 24 h, cyclin D1, Bcl-XL and Bcl2 were decreased significantly in both transcriptional (mRNA) and translational (protein) levels (Fig. 4c).

Next we constructed the STAT3-KNOCKDOWN T24 cell line (T24/STAT3-KD) and the normal control cell line (T24/control) to compare the metformin sensitivity between STAT3 inactivated and activated bladder cancer cells (Fig. 4e). Under normal culture conditions, T24/STAT3-KD cells proliferated less rapidly than T24/control cells (data not shown). As shown in Fig. 4f, metformin treatment for 48 h could suppress the proliferation of both cell lines, however, the cell viablity of T24/control cells was inhibited much more significantly than T24/STAT3-KD cells (*P* < 0.01). Then we evaluated the inhibitory effects on migration and invasion abilities of both cell lines after 5 mM metformin treatment. Normally T24/STAT3-KD cells had weakened migration and invasion abilities, compared with T24/control cells. Interestingly, metformin treatment was capable of decreasing T24/control cells by 237.7 ± 22.7 and 119.7 ± 6.8, in migration and invasion assays respectively. By constrast, such an inhibitory effect attenuated significantly in T24/STAT3-KD cells as migrating and invading cells were reduced by only 131.7 ± 19.8 (*P* < 0.01) and 87.7 ± 15.2 (*P* = 0.029), respectively (Fig. 4g).

Taken together, STAT3 played an important role for precancerous progression to invasive ones, metformin could efficiently inactivate STAT3, and as a result, transcription of its target genes was inhibited, leading to suppression of tumor development or tumorigenesis.

## Discussion

This study explored the anti-tumor effects of metformin on bladder cancer using both *in vitro* and *in vivo* models of human bladder cancers. We have found that metformin play an important role in blocking progression of precancerous lesions to invasive cancer in bladder, and this effect is mediated by inhibiting STAT3 phosphorylation, which leads to suppress a number of target genes of STAT3, such as cyclin D1, Bcl-XL and Bcl2. Therefore, metformin blocks the precancerous progression through inhibiting STAT3-mediated signal pathways involved in cell survival, proliferation, migration and invasion.

As an inexpensive and effective oral drug, metformin has been applied for the treatment of type 2 diabetes for decades. Its potential mechanisms are to promote glucose uptake and increase fatty acid oxidation in muscle and liver without obvious adverse effects [5, 16]. Although the value of metformin has been investigated in clinical trials for many types of cancers [6–8], including two recent retrospective studies on bladder cancer [9, 10], its specific effects on bladder cancers such as proliferation, viability, migration and invasion have not been adequately investigated *in vitro* and *in vivo*. Although a recent study demonstrated that metformin could inhibit the proliferation of bladder cancer cell lines and the growth of bladder cancer in a xenograft model [17], our study further elucidates the mechanisms underlying metformin suppressing bladder cancer proliferation, expansion, migration and invasion *in vitro* and *in vivo*. Importantly, we have for the first time demonstrated that metformin can prevent precancerous progression through inhibiting STAT3-mediated signaling pathways in a MNU-induced orthotopic rat bladder cancer model. This finding is of significance in clinic practice for the development of novel strategy for preventing progression of NMIBC to invasive cancers.

The underlying mechanisms how metformin inhibits cancer cells remain to be further elucidated, while the signaling pathways involved in cell proliferation, cell cycling and apoptotic cell death have been considered as potential pathways [18–20]. In this study, treating two bladder cancer cell lines T24 and J82, which were derived, respectively, from MIBC and NMIBC, with metformin for 24 h and 48 h inevitably resulted in cell cycle arrest and apoptotic cell death, consistently with the observations from other laboratories [18–20]. One recent study demonstrated that metformin could arrest bladder cancer cells in the G_0_/G_1_ phase with concomitant decreases in the expression of cyclin D1, CDK4 and E2F1 [17], which is verified again in our study. However, the ability of metformin to induce apoptotic cell death remained controversial. It has been showed that metformin could block the cell cycle of prostate cancer cells at the G_0_/G_1_ phase but did not induce apoptotic cell death [21]. The discrepancy between studies may be associated with variations in experimental conditions, tumor types and diversity of cell lines.

The mechanisms of metformin anti-tumor effects are considered to be associated with both indirect and direct effects on cancer cells. Metformin not only lowers circulating glucose concentration via an increase in muscle glucose uptake and suppression of hepatic gluconeogenesis, but also circulating insulin levels [6, 8]. Previous studies have shown that the type 1 insulin-like growth factor receptor is over expressed in bladder cancer, and high doses of human insulin and insulin glargine can promote bladder cancer cell proliferation *in vitro* [22, 23]. These results suggest that metformin could indirectly inhibit tumor development through decreasing glucose and insulin in circulation. The direct effects of metformin were firstly evaluated on breast cancer cells [24, 25], in which AMPK was primarily activated and mammalian target of rapamycin (mTOR) signaling and protein synthesis were subsequently reduced. Such mechanism may also apply to bladder cancer cells [17]. In our study, metformin directly inhibited the phosphorylation of STAT3, inactivating STAT3-mediated signaling pathways involved in cell proliferation, viability, migration and invasion. When STAT3 pathway was inactivated in T24/STAT3-KD cells, these bladder cancer cells, like T24 cells treated with metformin, turned to the phenotypes of weakened prolifearation, migration and invasion abilities. Accordingly, unlike T24/control cells with a high phosphorylated STAT3 levels, the inhibitory effects of metformin on T24/STAT3-KD cells were significantly attenuated. In our experiments, some of STAT3 classic target genes such as cyclin D1, Bcl2, Bcl-XL were all significantly down regulated upon metformin treatment. Cyclin D1 plays a vital role in promoting cell cycle G_1_/S phases transition [26, 27], consistently with our findings that the metformin-treated bladder cancer cells were tended to be arrested at G_0_/G_1_ phase and the number of S phase cells were greatly decreased. Previous reports have shown that the cyclin D1 was constitutively overexpressed in several human tumors including bladder cancer [28]. Thus, the down regulation of STAT3/cyclin D1 signaling can account for the cell cycle arrest in the G_0_/G_1_ stage and reduced G_1_/S phase transition in the bladder cancer cells treated with metformin. Moreover, Bcl2 and Bcl-XL, as pro-survival genes directly transcripted by STAT3 [11], were also repressed by metformin, suggesting that down-regulaiton of STAT3/Bcl2/Bcl-XL signaling pathways in metformin-treated bladder cancer cells caused increased apoptotic cell death.

In additon, STAT3 directly binds to the promoter of matrix metalloproteinase(MMP) 2 and upregulates its expression. It may also regulates activity of other MMP family members [29, 30], which were associated with tumor invasion. STAT3 also regulates cellular migration by modulating the activity of Rho and Rac [31, 32]. In an orthotopic mouse bladder cancer model, STAT3-transgenic mice, compared with the wild-type counterparts, developed invasive cancer directly from carcinoma in situ (CIS) in a shorter time, bypassing the noninvasive papillary tumor stage [13]. These findings suggested that STAT3 is also a key regulator for migration and invasion of cancer cells and might play a master role in driving precancerous progression to invasive or malignant tumors. The hypothesis is supported by our findings that metformin treatment suppresses invasion and migration of bladder cancer cells *in vitro* and blocks precancerous lesions and CIS progressing to invasive cancers in orthotopic rat bladder cancer model. In consistence with the phenomenon, STAT3 phosphorylation was remarkably inhibited by metformin either *in vitro* or *in vivo* models of bladder cancers, accompanied by reduced capacity of migration and invasion of cancer cells *in vitro* and arrested progression of precancerous lesions. Moreover, the tumor developments in MNU-induced rats were arrested at the papillary tumor stage when treated with metformin. Therefore, we conclude that metformin can block precancerous progression to invasive cancers through inhibiting STAT3-mediated signaling pathways.

MNU-induced rat bladder cancer intimately mimicked human bladder cancer. Six weeks of MNU intravesical induction followed by 8 weeks of observation revealed all the stages of bladder cancer in pathology: bladder epithelial cells progressing from atypical hyperplasia to flat CIS and transitional cell carcinoma [33]. In our study, tumor incidence reached 100 %, consistently with previous reports [33, 34], but not with other study, in which the tumor incidence was much lower [35]. The tumor incidence was slightly reduced in metformin-treated group (87.5 %), suggesting metformin has the potential to inhibit cell transformation induced by MNU, but very limited. Essentially, metformin has little ability to inhibit uroepithelial transformation, but could almost completely block progression of preneoplasms or non-invasive tumors, because no invasive cancer and sarcoma was observed in metformin-treated rats and the tumors in metformin-treated group were limited at the stages of dysplasia, papillary tumor or CIS. Compared with the xenograft model of bladder cancer [17], our orthotopic rat bladder cancer model more realistically imitated the developmental process of human bladder cancer [33].

Moreover, feeding rats 2 g/L metformin solution means that each rat was administrated metformin at approximate 200 mg/kg/day, which has been reported as the no observable adverse effect level (NOAEL) in rats [36]. Accordingly, such doses of metformin caused no side-effect and body weight change in these rats (data not shown). Considering the information that the maximum recommended daily dose of metformin for the treatment of type 2 diabetic patients is 2550 mg/day and the fact that 200 mg/kg/day in rats is equivalent to 1200 mg/60 kg/day in humans, our study suggested that metformin could inhibit bladder cancer in vivo even at a safe daily dose.

It has been considered that tumor development is mediated by tumor stem cells (TSCs) [37–39]. A TSC can be initiated from a healthy cell that hierarchically develops into tumor-initiating stem cell (TISC), precancerous stem cell (pCSC) and then cancer stem cell (CSC) in carcinogenic environments. The progenies of CSCs, cancer-propagating progenitor cells (CPCs), are a major population contributing to the formation of tumor mass [40]. Although this novel model for TSC development has attracted the attentions from colleagues, it remains to be verified in animals and human. The MNU-induced orthotopic rat bladder cancer model is suitable for verification of the TSC model, and metformin may be used to precisely dissect or demarcate between precancerous and cancerous stages of a bladder TSC.

In summary, this study demonstrates that metformin can block preneoplasm or non-invasive cancer of bladder progressing to invasive cancer through inhibiting STAT3-mediated signaling pathways which can promote viability, proliferation, migration and invasiveness of cancer cells. Our results suggest that metformin could be a potential candidate for the development of novel therapeutic strategies for human bladder cancer.

## Additional file

Additional file 1: Figure S1. Animal experiment design.
