# Supplementary figures and images for: Metformin can block precancerous progression to invasive tumors of bladder through inhibiting STAT3-mediated signaling pathways
Source: J Exp Clin Cancer Res. 2015 Aug 7;34(1):77. doi: 10.1186/s13046-015-0183-0 (PMC4553001; doi:10.1186/s13046-015-0183-0)

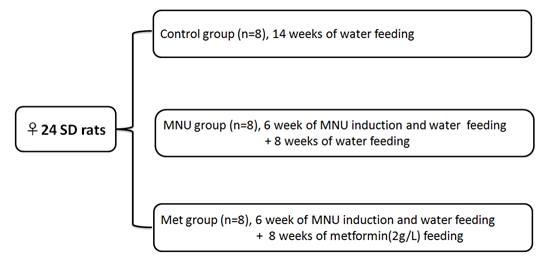

Supplement: Additional file 1: Figure S1. — Animal experiment design. [file 13046_2015_183_MOESM1_ESM.tif]
